# Intake of Ultra-Processed Food and Ectopic-, Visceral- and Other Fat Depots: A Cross-Sectional Study

**DOI:** 10.3389/fnut.2022.774718

**Published:** 2022-04-04

**Authors:** Michael Fridén, Joel Kullberg, Håkan Ahlström, Lars Lind, Fredrik Rosqvist

**Affiliations:** ^1^Department of Public Health and Caring Sciences, Clinical Nutrition and Metabolism, Uppsala University, Uppsala, Sweden; ^2^Department of Surgical Sciences, Radiology, Uppsala University, Uppsala, Sweden; ^3^Antaros Medical AB, BioVenture Hub, Mölndal, Sweden; ^4^Department of Medical Sciences, Clinical Epidemiology, Uppsala University, Uppsala, Sweden

**Keywords:** ultra-processed food, diet, liver fat, ectopic fat, visceral adipose tissue (VAT)

## Abstract

**Introduction:**

The purpose of this study was to investigate associations between intake of ultra-processed food (UPF) and liver fat, pancreas fat and visceral adipose tissue (VAT) but also subcutaneous adipose tissue (SAT), VAT/SAT ratio and total fat mass.

**Materials and Methods:**

Cross-sectional analysis of *n* = 286 50-year old men and women. Energy percentage (%E) from UPF was calculated from a semi-quantitative food frequency questionnaire. Food items were categorized according to the NOVA-classification system and fat depots were assessed using magnetic resonance imaging (MRI) and bioelectrical impedance analysis (BIA). Associations were analyzed using linear regression, adjusted for sex, education, physical activity, smoking, dietary factors and BMI.

**Results:**

Mean intake of UPF was 37.8 ± 10.2 %E and the three largest contributors to this were crisp- and wholegrain breads and spreads, indicating overall healthy food choices. Consumption of UPF was associated with higher intake of energy, carbohydrates and fiber and lower intake of protein and polyunsaturated fat but no differences were observed for total fat, saturated fat (SFA), monounsaturated fat, sugar or alcohol between tertiles of UPF. Intake of UPF was positively associated with liver- and pancreas fat, VAT, VAT/SAT and inversely associated with total fat mass in crude models. The association for VAT remained after full adjustment (β = 0.01 (95% CI: 0.002, 0.02), *P* = 0.02) and was driven by women.

**Conclusion:**

Energy intake from UPF is not associated with ectopic fat, SAT or total fat after adjustment for multiple confounders in this population having overall healthy food habits. However, a positive association between UPF and VAT was observed which was driven by women.

## Introduction

Ultra-processed food (UPF), defined as “formulations made mostly or entirely from substances derived from foods and additives” (1), has increased markedly over the past 50 years, in parallel with the rise in obesity prevalence (2, 3). In Sweden alone, consumption of UPF increased by 142% between 1960 and 2010, which was largely driven by an increase in soda consumption (+315%) and snack foods such as crisps and candies (+367%) (2). Although UPFs are cheap, convenient and contribute to some important nutrients, they are generally more energy-dense and less nutrient-dense, higher in added sugar and saturated fat (SFA) and lower in fiber (4–6). Unsurprisingly therefore, intake of UPF has consistently been associated with several lifestyle diseases such as cardiovascular disease (CVD) and type-2 diabetes mellitus (T2DM) (7–10), diseases which have been proposed to be causally linked with accumulation of ectopic fat (e.g., liver fat and pancreas fat) and visceral fat mass (11, 12). Whether associations observed between intake of UPF and CVD and T2DM are mediated by ectopic fat mass and visceral adipose tissue (VAT) warrants further investigation. Interestingly, nutrients closely related to UPF, such as SFA, added sugar and fiber have been associated with ectopic fat, in particular liver fat and liver-specific diseases such as non-alcoholic fatty liver disease (NAFLD) (13–18). However, associations between intake of UPF and liver- and pancreas fat has not yet been investigated and to our knowledge, only one study examining the link between UPF and VAT has so far been published (19). Furthermore, studies on the associations between intake of UPF and other fat depots such as subcutaneous adipose tissue (SAT), the ratio of VAT to SAT and total fat mass are limited. We therefore conducted a cross-sectional study to examine these associations, with focus on liver fat, in a middle-aged Swedish population. We hypothesized that consumption of UPF would be positively associated with liver fat and all other fat depots.

## Materials and Methods

### Subjects

This cross-sectional study is based on baseline-data from a prospective population-based cohort study initiated in Uppsala in 2010, named POEM (Prospective investigation of Obesity, Energy and Metabolism). Participants were invited via mail after their 50th birthday and thereafter signed a written informed consent if they accepted to participate. A total of *n* = 502 individuals (out of *n* = 2008) accepted the invitation. The study was approved by the Ethics Committee of Uppsala University (Uppsala Dnr 2009/057 and Dnr 2012/143) and was conformed to the ethical guidelines of the 1964 Declaration of Helsinki and its later amendments. A more detailed description of the POEM-cohort study has previously been presented (20).

Individuals with complete data on liver fat and diet constituted the study population of this analysis. Those with implausible energy intake (<600 kcal/day and >3,500 kcal/day for women and <800 kcal/day and >4,200 kcal/day for men) as well as extreme outliers [±1.5 times the interquartile range (IQR)] of UPF consumption were excluded (*n* = 28). The final population sample consisted of *n* = 286 50-year old men and women.

### Diet Assessment and Classification of UPF

UPF, expressed as percentage of total energy intake (%E), was the main exposure in this study. A semi-quantitative food frequency questionnaire (FFQ) consisting of 140 items (including alcohol) was provided to all participants at baseline in order to capture habitual dietary intake over the past year. Diet exposure was either categorized into eight frequency categories ranging from zero times/month to three+ times/day or presented with open answers where participants had to fill in how many slices/table spoons/cups/glasses per week or day they consumed of the food item. Nutrient composition was calculated using age-specific portion sizes and the Swedish Food Composition Database. A shorter version of the same FFQ that was used in this study has been validated against subcutaneous adipose tissue fatty acid biomarkers and weighed food records (21, 22).

UPFs were classified according to the NOVA-classification system (1, 23). NOVA classifies food items into four categories depending on the degree and purpose of industrial processing: unprocessed or minimally processed foods (NOVA 1) (e.g., broccoli and salmon), processed culinary ingredients (NOVA 2) (e.g., oil and sugar), processed foods (NOVA 3.1) (e.g., canned tuna and fried potatoes) and ultra-processed foods (NOVA 3.2) (e.g., sugar-sweetened beverages and breakfast cereals with added sugar) (1, 23). Food items from the FFQ were independently classified into the four food categories by M.F. and F.R. A follow-up discussion took place upon disagreement to determine the correct category of which the food item should be classified. Butter/margarine as bread spread was categorized as UPF since they were difficult to separate and nearly all participants consumed either a mixture of butter and margarine or solely margarine on their bread. The energy intake from all UPFs were added and then divided by the total energy intake.

An additional score called the healthy diet indicator (HDI) was calculated to reflect the overall quality of the diet. This score is based on the adherence to the World Health Organization (WHO) nutrition guidelines and was first introduced by Huijbregts et al. in (24). Due to changes in these guidelines over time, a revised HDI score was used in this study (25). A total score of maximum eight points from eight food and nutrient categories (SFA, PUFA, fiber, protein, cholesterol, total sugar (monosaccharides and disaccharides), sodium intake and fruits and vegetables) was calculated. Subjects received one point if SFA was <10 %E, PUFA was between 6 and 10 %E, fiber was >25 g, protein was between 10 and 15 %E, cholesterol was <300 mg, total sugar was <10 %E, sodium was <2,000 mg and fruits and vegetables was ≥400 g.

### Ectopic Fat, VAT and Other Fat Depots

Liver fat was determined a priori to be the primary outcome of interest in this study, while pancreas fat and VAT were secondary outcomes. SAT, total fat mass and the ratio of VAT to SAT were included as exploratory outcomes. Ectopic fat depots, VAT and SAT were assessed using a 1.5T clinical magnetic resonance imaging (MRI) system (Achieva, Philips Healthcare, Best, Netherlands). The methodology of MRI and quantification of ectopic fat, VAT and SAT have been described in detail (26). Total fat mass was assessed using bioelectrical impedance analysis (BIA) (Tanita BC-418, Tokyo, Japan). Due to missing information on competing risk factors for NAFLD (e.g., viral hepatitis) and due to the fact that only one male individual consumed excess alcohol (>30 grams/day) and had a liver fat content exceeding 5.5%, prevalence of NAFLD (yes/no) was determined solely based on a hepatic fat content exceeding 5.5%.

### Sociodemographic and Lifestyle Variables and Anthropometrics

Covariates included in the multivariable models were chosen based on (1) that previous studies on UPF and cardiometabolic diseases had included them as covariates (sex, education, current smoking status and physical activity) (7, 9) and/or (2) that previous studies demonstrated an effect or an association with liver fat and UPF (3–6, 13–18, 25). Model 1 included sex (male/female), education (9 years, 10–12 years, University), current smoking status (yes/no) and physical activity (sedentary, light, moderate, high) as categorical variables. Model 2 included covariates from model 1 + continuous dietary intake data (obtained from FFQ) that have been shown to be associated with both liver fat and UPF (4–6, 13–18, 27). These nutrients were protein (%E), alcohol (%E), SFA (%E), polyunsaturated fat (PUFA) (%E), fiber (g) and total sugar (monosaccharides + disaccharides) (%E). Model 3 included covariates from model 1 and model 2 + body mass index (BMI). BMI was calculated as the weight in kilograms (kg) divided by the height in meters (m) squared and was included in the last model as a potential mediator between UPF and fat depots. BMI was treated as a continuous variable. As for physical activity, two questions were posed to the participants: (1) how many times/week do you engage in light physical activity for at least 30 min? (2) how many times/week do you engage in moderate to intense physical activity for at least 30 min (you should be sweaty)? From these two questions, levels of physical activity were derived: level 1 (sedentary) corresponding to <2 times/week of light activity and 0 times/week of moderate to intense activity; level 2 (light) corresponding to >1 times/week of light activity and 0 times/week of moderate to intense activity; level 3 (moderate) corresponding to 1–2 times/week of moderate to intense activity and; level 4 (high) corresponding to >2 times/week of moderate to intense activity.

### Clinical Variables

Fasting plasma glucose, serum insulin, total cholesterol, low-density lipoprotein (LDL)-cholesterol, high-density lipoprotein (HDL)-cholesterol and triglycerides (TG) were measured after an overnight fast and analyzed using routine laboratory techniques at Uppsala University Hospital. Homeostatic Model Assessment of Insulin Resistance (HOMA-IR) was calculated using the following formula: fasting plasma glucose (mmol/L) x fasting serum insulin (mU/L) divided by 22.5 (28).

### Statistical Analysis

The primary analysis of this study was to investigate the association between intake of UPF and liver fat, while secondary analyses included the associations between intake of UPF and pancreas fat and VAT. SAT, VAT/SAT and total fat mass were included as exploratory outcomes. UPF and fat depots were treated as continuous variables. Normal distribution was assessed using the Shapiro-Wilk W test and when appropriate, skewed distributed variables were logarithmically transformed before analysis (BMI, fiber, PUFA, total sugar and alcohol for the primary analysis and BMI, PUFA, total sugar and alcohol for the subgroup analysis). Simple and multivariable linear regression analyses were performed between UPF and ectopic fat depots, VAT, SAT, VAT/SAT and total fat mass. Multivariable models included sex and current smoking status as nominal variables; education and physical activity as ordinal variables; and protein, alcohol, SFA, PUFA, fiber, total sugar and BMI as continuous variables. Sex-stratified multivariable models were performed as *post hoc* analyses. Sensitivity analyses excluding whole-grain bread and crisp bread from the multivariable models were performed. These analyses were conducted due to two reasons. The first reason alluded to the fact that whole-grain bread and crisp bread could likewise be categorized as processed food items (NOVA 3.1) depending on if any sugar, oil or additives have been added. The second reason was due to the fact that these food items contributed 23.0 ± 14.7 % of the energy coming from UPF (data not shown). A variance inflation factor (VIF) >5 was set a priori as the cut-off for demonstrating multicollinearity.

A one-way analysis of variance (ANOVA) comparing dietary nutrient composition and HDI scores between tertiles of UPF-intake was conducted as an exploratory analysis. Two-sample *t*-tests were performed to compare variables of interest between end-tertiles of UPF-intake in the full population sample and separately for men and women. Crude and multivariable logistic regression was used to calculate odds ratios and 95% confidence intervals (CI) for the prevalence of NAFLD, with UPF (%E) included as the independent continuous variable. The multivariable logistic regression model included sex and current smoking status as nominal variables; education and physical activity as ordinal variables; and protein, alcohol, SFA, PUFA, fiber, total sugar and BMI as continuous variables. Categorical variables were compared using chi-squared tests. Non-parametric tests (e.g., Kruskal-Wallis-, Mann-Whitney U- and Fisher's exact tests) were performed when the underlying assumptions of the equivalent parametric statistical test were not satisfied. Levene's test was used to assess homogeneity of variance between groups. No correction for multiple comparisons was applied. All statistical analyses were performed using JMP software version 15.1.0 (SAS Institute, Inc) and a *P*-value <0.05 was set as the significance level.

## Results

Population characteristics are presented in Table 1. The study population of equally distributed 50-year old men and women had a median BMI of 25.8 (23.4–28.6) kg/m^2^, a median fasting plasma glucose of 5.0 (4.8–5.3) mmol/L and a mean LDL-cholesterol of 3.4 ± 0.9 mmol/L. Approximately half (49%) had a University degree and 7.7% were current smokers. UPF contributed to 37.8 ± 10.2 %E while minimally processed foods contributed to 43.7 ± 10.6 %E.

**Table 1:** Characteristics of the study population (*n* = 286)^a^.

	**All (*n =* 286)**	**T1 of UPF (*n =* 95)^b^**	**T2 of UPF (*n =* 95)**	**T3 of UPF (*n =* 96)**	***P*-value (T3 vs T1)**
Sex (% women/men)	53/47	75/25	56/44	30/70	*P* < 0.0001
Education (% 9y/10–12y/university)	5/46/49	2/42/56	7/46/46	6/49/45	*P =* 0.13^**^
Physical activity (% sedentary/light/moderate/high)	13/24/32/31	9/14/35/42	13/27/31/30	17/31/31/21	*P =* 0.001
Current smoking (%)	7.7	8	8	6	*P =* 0.56
BMI (kg/m^2^)	25.8 (23.4–28.6)	25.4 (22.4–28.6)	26.0 (23.8–28.5)	26.0 (24.0–29.6)	*P =* 0.09^*^
Liver fat (%)	2.1 (1.1–5.1)	1.5 (0.9–3.1)	2.3 (1.1–5.5)	2.4 (1.3–6.1)	*P =* 0.01
NAFLD prevalence (n/%)	66/23	18/19	23/24	25/26	*P =* 0.24
VAT (L)	3.2 (1.7–4.5)	2.0 (1.2–3.6)	3.5 (1.8–5.0)	3.7 (2.3–5.4)	*P* < 0.0001^*^
Pancreas fat (%)	4.3 (2.4–7.2)	3.0 (2.0–6.0)	4.3 (2.6–7.2)	5.1 (2.9–8.1)	*P =* 0.007
SAT (L)	6.3 (4.5–8.4)	5.9 (3.9–8.7)	6.4 (5.2–8.4)	5.8 (4.2–8.3)	*P =* 0.97
VAT/SAT	0.4 (0.3–0.7)	0.3 (0.2–0.5)	0.4 (0.3–0.8)	0.6 (0.4–0.9)	*P* < 0.0001
Total fat mass (%)	28.4 ± 8.7	30.3 ± 8.9	29.0 ± 8.0	25.9 ± 8.7	*P =* 0.0006
Fasting plasma glucose (mmol/L)	5.0 (4.7–5.3)	5.0 (4.8–5.3)	4.9 (4.7–5.3)	5.0 (4.6–5.4)	*P =* 0.63^*^
Fasting serum insulin (mU/L)	3.6 (2.4–5.9)	2.9 (1.9–5)	4.2 (2.8–6)	3.9 (2.9–6.6)	*P =* 0.001^*^
HOMA-IR	0.8 (0.5–1.3)	0.6 (0.4–1.1)	0.9 (0.6–1.3)	0.9 (0.6–1.5)	*P =* 0.007
Total cholesterol (mmol/L)	5.3 ± 1.0	5.4 ± 1.0	5.2 ± 1.0	5.2 ± 0.8	*P =* 0.21
LDL-cholesterol (mmol/L)	3.4 ± 0.9	3.4 ± 0.9	3.3 ± 0.9	3.4 ± 0.8	*P =* 0.96
HDL-cholesterol (mmol/L)	1.4 (1.2–1.6)	1.5 (1.3–1.7)	1.4 (1.1–1.5)	1.3 (1.1–1.5)	*P* < 0.0001
TG (mmol/L)	1.0 (0.7–1.4)	0.8 (0.7–1.3)	1.0 (0.8–1.6)	1.1 (0.8–1.4)	*P =* 0.02
NOVA group 1 (%E)	43.7 ± 10.6	52.6 ± 9.8	43.7 ± 6.7	34.8 ± 6.4	*P* < 0.0001
NOVA group 2 (%E)	0.0 (0.0–0.4)	0.0 (0.0–0.3)	0.0 (0.0–0.6)	0.0 (0.0–0.2)	*P =* 0.75
NOVA group 3.1 (%E)	18.1 ± 7.4	20.4 ± 8.9	18.1 ± 6.6	15.8 ± 5.6	*P =* 0.0004^*^
NOVA group 3.2 (%E)	37.8 ± 10.2	26.6 ± 5.0	37.6 ± 2.6	49.0 ± 5.4	*P* < 0.0001

a *Data are presented as mean ± SD, %, counts or as median (IQR) for skewed distributed variables*.

b *Tertile 1 indicates the lowest proportion of UPF-intake while tertile 3 indicates the highest proportion of UPF-intake*.

*BMI, Body Mass Index; HDL, High-Density Lipoprotein; HOMA-IR, Homeostatic Model Assessment of Insulin Resistance; LDL, Low-Density Lipoprotein; NAFLD, Non-alcoholic fatty liver disease; T, Tertile; TG, Triglycerides; UPF, Ultra-processed food; VAT, Visceral Adipose Tissue; NOVA group 1, unprocessed or minimally processed foods; NOVA group 2, processed culinary ingredients; NOVA group 3.1, processed foods; NOVA group 3.2, ultra-processed foods*.

* *Analyzed non-parametrically using the Mann-Whitney U-test*.

** *9y + 10–12y categories were combined to satisfy the assumption of the chi-squared test (at least 80% of the expected frequencies are greater than or equal to 5)*.

The contribution of energy from UPF among tertiles were: 26.6 ± 5.0 %E (tertile 1), 37.6 ± 2.6 %E (tertile 2) and 49.0 ± 5.4 %E (tertile 3). The highest tertile of UPF consumption had more men and was less physically active compared to the lowest tertile. In addition, compared to the lowest tertile, the highest tertile had significantly higher liver fat [2.4 (1.3–6.1) % vs. 1.5 (0.9–3.1) %], pancreas fat [5.1 (2.9–8.1) % vs. 3.0 (2.0–6.0) %], VAT [3.7 (2.3–5.4) L vs. 2.0 (1.2–3.6) L], VAT/SAT [0.6 (0.4–0.9) vs. 0.4 (0.3–0.7)., fasting serum insulin [3.9 (2.9–6.6) mU/L vs. 3.6 (2.4–5.9) mU/L], HOMA-IR [0.9 (0.6–1.5) vs. 0.8 (0.5–1.3)] and TG [1.1 (0.8–1.4) mmol/L vs. 0.8 (0.7–1.3) mmol/L] but lower HDL-C [1.3 (1.1–1.5) mmol/L vs. 1.5 (1.3–1.7) mmol/L] and total fat mass (25.9 ± 8.7 % vs. 30.3 ± 8.9 %). Differences in liver fat, VAT, VAT/SAT, fasting serum insulin, HOMA-IR and TG between end-tertiles of UPF intake were only statistically significant in women but not in men. The contribution of energy from UPF in men were: 42.0 ± 9.5 %E and in women: 34.1 ± 9.3 %E. (Supplementary Table 1).

### UPF and Nutrient Composition

When intake of UPF, expressed as %E, was categorized into tertiles, reported energy-, carbohydrate- and fiber intake were higher, while protein and PUFA were lower in the highest tertile of UPF compared to the lowest tertile. Energy, carbohydrate, protein and fiber intake also differed between tertile 3 and tertile 2 of UPF. Reported intake of total fat, SFA, monounsaturated fat (MUFA), total sugar and alcohol did not differ between tertiles. HDI scores differed between tertile 3 and tertile 1 (1.8 ± 1.0 vs. 2.4 ± 1.2, *P* = 0.0002) as well as between tertile 2 and tertile 1 (2.0 ± 1.2 vs. 2.4 ± 1.2, *P* = 0.02) (Table 2). Intakes of carbohydrates and protein were similar among UPF-tertiles in both men and women whereas energy intake was higher among tertiles in men only and consumption of PUFA was lower among tertiles in women only (Supplementary Table 1).

**Table 2:** Nutrient composition between tertiles of ultra-processed food (UPF)^a^.

	**UPF T1 (*n =* 95)^b^**	**UPF T2 (*n =* 95)**	**UPF T3 (*n =* 96)**	**Between all groups^c^**	**T3 vs. T1**	**T3 vs. T2**	**T2 vs. T1**
Energy (kcal)	2041.4 ± 593.7	2200.4 ± 654.8	2566.3 ± 705.5	*P* < 0.0001	*P* < 0.0001	*P* = 0.0001	*P* = 0.09
Carbohydrates (%E)^*^	39.3 ± 8.1	41.5 ± 5.6	44.3 ± 5.4	*P* < 0.0001	*P* < 0.0001	*P* = 0.001	*P* = 0.03
Fiber (g)	26.6 (19.5–31.4)	25.3 (19.9–32.9)	31.0 (23.0–38.1)	*P* = 0.001	*P* = 0.0009	*P* = 0.002	*P* = 0.83
Total sugar (%E)	15.1 (12.3–18.3)	14.4 (12.3–17.3)	14.9 (12.1–16.8)	*P* = 0.55	-	-	-
Fat (%E)^*^	35.0 ± 7.2	34.8 ± 4.9	33.4 ± 4.3	*P* = 0.17	-	-	-
SFA (%E)^*^	14.4 ± 3.6	14.7 ± 3.1	14.1 ± 2.6	*P* = 0.57	-	-	-
MUFA (%E)^*^	11.6 (10.6–13.3)	11.8 (11.1–12.6)	11.4 (10.0–12.6)	*P* = 0.15	-	-	-
PUFA (%E)^*^	5.5 (4.3–6.5)	4.9 (4.5–5.8)	4.9 (4.3–5.6)	*P* = 0.02	*P* = 0.007	*P* = 0.32	*P* = 0.06
Protein (%E)^*^	20.0 ± 2.9	18.2 ± 2.1	17.3 ± 1.9	*P* < 0.0001	*P* < 0.0001	*P* = 0.003	*P* < 0.0001
Alcohol (%E)	2.4 (1.1–4.0)	2.5 (1.1–3.7)	1.9 (0.7–3.4)	*P* = 0.18	-	-	-
HDI	2.4 ± 1.2	2.0 ± 1.2	1.8 ± 1.0	*P* = 0.0009	*P* = 0.0002	*P* = 0.19	*P* = 0.02

a *Data are presented as mean ± SD or as median (IQR) for skewed distributed variables*.

b *Tertile 1 indicates the lowest proportion of UPF-intake while tertile 3 indicates the highest proportion of UPF-intake*.

c *For statistically significant mean/distributional differences between groups (P < 0.05) using one-way ANOVA or Kruskal-Wallis tests, further post-hoc tests for pair-wise comparisons were conducted*.

*HDI, healthy diet indicator; MUFA, monounsaturated fat; PUFA, polyunsaturated fatty acids; SFA, saturated fatty acids; T, tertile; UPF, ultra-processed food*.

* *Analyzed non-parametrically using the Kruskal-Wallis test*.

Food items that contributed to energy in the UPF-category were, in descending order of reported median energy intake: crisp bread, whole-grain bread, butter/margarine spread, pizza, chocolate, buns/cookies, fiber-enriched bread, pancakes/crepes, chips/popcorn/cheese puffs, other sausage, sausage, cakes/pastries, ice cream, white bread, muesli, candy, mayonnaise, biscuits/wafers, French fries, other jam, cold cuts sausage, lingonberry jam, salad dressing, Swedish caviar, ketchup, spirits, fruit yogurt/sour milk, blood pudding/sausage, pea soup, liver paté, other soda/fruit juice, breakfast cereal, lean sausage, liqueur/sherry, salad dressing (reduced fat/fat free), Coca Cola/Pepsi light, Coca Cola/Pepsi, other soda/fruit juice light, cream cheese (low-fat), cream cheese, liver paté (low-fat), fish sticks, fruit fool/fruit soup and mayonnaise (reduced fat/fat free) (Supplementary Table 2). The three largest contributors to UPF (crisp bread, whole-grain bread and butter/margarine spread) were similar between men and women (Supplementary Table 2).

### UPF and Ectopic-, Visceral- and Other Fat Depots

In crude linear regression models, intake of UPF was associated with ln liver fat (β = 0.02, *P* = 0.006), ln pancreas fat (β = 0.01, *P* = 0.01), ln VAT (β = 0.02, *P* < 0.0001), ln VAT/SAT (β = 0.02, *P* < 0.0001) and total fat mass (β = −0.2, *P* < 0.0001) (Table 3). However, after adjustment for sex, education, physical activity and current smoking status in model 1, all associations were attenuated. These associations remained non-statistically significant when further adjusting for dietary factors in model 2 and BMI in model 3, except for VAT that turned statistically significant in model 2 (β = 0.01, *P* = 0.04), which also remained in model 3 (β = 0.009, *P* = 0.02).

**Table 3:** Linear regression analyses between ultra-processed food (%E) and fat depots^a^.

	**Crude β (95% CI)**	***P***	**Model 1^b^ β (95% CI)**	***P***	**Model 2^c^ β (95% CI)**	***P***	**Model 3^d^ β (95% CI)**	***P***
Ln liver fat	0.02 (0.005, 0.03)	0.006	−0.003 (−0.02, 0.01)	0.57	0.01 (−0.01, 0.02)	0.39	0.005 (−0.01, 0.02)	0.47
Ln pancreas fat	0.01 (0.003, 0.02)	0.01	0.002 (−0.01, 0.01)	0.71	0.01 (−0.004, 0.02)	0.16	0.01 (−0.005, 0.02)	0.26
Ln VAT	0.02 (0.01, 0.03)	<0.0001	0.004 (−0.005, 0.01)	0.35	0.01 (0.001, 0.02)	0.04	0.01 (0.002, 0.02)	0.02
Ln SAT	−0.0001 (−0.01, 0.01)	0.97	0.0002 (−0.01, 0.01)	0.96	0.005 (−0.003, 0.01)	0.22	0.003 (−0.001, 0.01)	0.16
Ln VAT/SAT	0.02 (0.02, 0.03)	<0.0001	0.004 (−0.001, 0.01)	0.13	0.01 (−0.0005, 0.01)	0.07	0.01 (−0.001, 0.01)	0.08
Total fat mass	−0.20 (−0.30, −0.10)	<0.0001	−0.04 (−0.12, 0.05)	0.38	−0.03 (−0.13, 0.07)	0.55	−0.05 (−0.11, 0.01)	0.10

a *Data are presented as β with 95% CI and corresponding P-values*.

*SAT, subcutaneous adipose tissue; VAT, visceral adipose tissue*.

*n (liver fat) = 282–286, n (pancreas fat) = 276–279 , n (VAT, SAT, VAT/SAT) = 238–241, n (Total fat mass) = 281–285*.

b *Adjusted for sex, education, physical activity and current smoking status*.

c *Adjusted for Model 1 + protein, fiber, alcohol, saturated fat, polyunsaturated fat and total sugar*.

d *Adjusted for Model 2 + BMI (Body mass index)*.

Using binary logistic regression to model NAFLD prevalence (yes/no) with UPF expressed as %E, the odds ratio with a 95% CI per 10 %E change in intake of UPF was: 1.27 (95% CI, 0.97-1.66), P = 0.09 in the crude model. When further adjusting for potential confounders in a multivariable model, the relationship was attenuated [odds ratio: 1.32 (95% CI, 0.84–2.09), *P* = 0.23)].

*Post hoc* sensitivity analyses with whole-grain bread and crisp bread excluded from the UPF-category (NOVA group 3.2) demonstrated similar associations between intake of UPF and ectopic fat depots, VAT, SAT, VAT/SAT and total fat mass as the main model presented in Table 3. Some minor discrepancies were observed for liver fat (stronger association in *post hoc* analyses) and for VAT (precision of the estimate wider in *post hoc* analyses) (Supplementary Table 3). Excluding whole-grain bread and crisp bread from the UPF category changed the proportion of UPF to total energy intake from 37.8 ± 10.2 %E to 29.0 ± 9.7 %E.

Further *post hoc* analyses included sex-stratified multivariable linear regression, demonstrating similar results as the non-stratified main models (Supplementary Table 4). Associations between UPF and VAT (β = 0.01 (95% CI, 0.003–0.02), *P* = 0.01) and VAT/SAT [β = 0.01 (95% CI, 0.003–0.02), *P* = 0.01] were statistically significant in women but not in men in the fully adjusted model.

## Discussion

In this cross-sectional study, intake of UPF, as defined by the NOVA classification system, is positively associated with liver- and pancreas fat, VAT and VAT/SAT and inversely associated with total fat mass in crude analyses. However, when adjusting for potential confounders such as sex, education, physical activity and current smoking status, all associations are attenuated and most of them no longer statistically significant. Only the positive association between UPF and VAT remains significant after further adjustments for dietary factors and BMI and sex-specific analyses revealed that this is driven by women.

Our finding for liver fat is in accordance with a recently published randomized cross-over feeding trial, where *n* = 20 participants consumed either UPF or minimally processed food *ad libitum*, matched for nutrients such as total sugar and fiber, for 2 weeks and thereafter crossed over to the other diet for 2 additional weeks (29). Liver fat was assessed in a subgroup of *n* = 13 using magnetic resonance spectroscopy (MRS). Although participants consumed more than 500 extra kcal per day on the UPF-diet (primary end point), liver fat did not differ between diet periods. Interestingly, intake of total sugar and fiber (among other nutrients) did not differ between diets in this study, which may partially explain the null findings. Neither total sugar nor SFA intake differed between tertiles of UPF in our study. Since SFA in particular, and to a lesser extent added sugar, have shown to increase liver fat in eucaloric and hypercaloric settings (13–16, 18), the lack of difference in these nutrients may partially explain the weak associations between UPF and liver fat in crude models and the lack of associations in adjusted models. Furthermore, fiber intake differed between tertiles and if expressed in absolute terms of grams, the highest tertile of UPF actually reported a higher median fiber intake compared to the lowest tertile [31.0 (23.0–38.1) gram vs. 26.6 (19.5–31.4) gram, *P* = 0.0009] (Table 2), likely due to the higher intake of crisp- and whole-grain breads. These results could also have contributed to our findings since fiber intake has been inversely associated with NAFLD in previous observational studies (17). However, despite higher intake of fiber in tertile 3 of UPF intake and similar intake of SFA and total sugar between end-tertiles, the overall quality of the diet, as assessed by HDI scores, was lower in tertile 3, although not clinically significant. HDI-scores among tertiles were generally low, but somewhat in line with other population-based cohorts in Sweden (30). Finally, in accordance with the cross-over trial, our study also showed a 500 kcal difference in energy intake between tertiles of UPF intake. Accumulation of some ectopic fat depots (e.g., liver fat) may be more responsive to changes in specific nutrients (such as SFA, fiber and total sugar) than for energy intake per se, as has previously been suggested (14–16, 18) and warrants further investigation. In contrast, intake of UPF was recently demonstrated to be associated with the development of NAFLD (assessed by ultrasonography) over 4.2 years in a Chinese cohort (31). These associations remained in models adjusting for multiple potential confounders, including overall diet quality, but unfortunately not carbohydrate- or fat composition (e.g., sugars, fiber, SFA). These discordant findings could be due to various factors (e.g., method for assessing liver fat/NAFLD, different populations, study designs and consumption patterns of UPF) and results should be compared cautiously.

UPF is a highly heterogeneous food category and the association between UPF and liver fat needs to be investigated in other geographical regions where food items within the UPF-category may differ from food items consumed in Uppsala, Sweden. While crisp- and whole-grain bread and butter/margarine provided almost a third of the energy from UPF (30.6 ± 16.0 %E), indicating a generally healthy dietary pattern, these findings are in stark contrast to findings from other countries such as Australia, Mexico and the United Kingdom (4–6). The three largest contributors to energy from UPF in these populations were either mass-produced packaged breads, frozen and shelf stable ready meals and fast food dishes (Australia); cookies, pastries and sweet bread, carbonated sugar-sweetened beverages and salty snacks (Mexico); or industrialized packaged bread, packaged pre-prepared meals and breakfast cereals (the United Kingdom). Importantly, an increase in absolute energy intake from crisp- and whole-grain bread and butter/margarine spread was also noticeable between tertiles of UPF (data not shown). Evidently, the type of UPF in our population differs from the type of UPF in other countries where associations between UPF and health outcomes have previously been investigated. This apparent difference is also demonstrated in a newly published study from Spain where associations between UPF and VAT and total fat mass (but not liver fat) were prospectively analyzed using data from the PREDIMED-Plus trial (19). Although a positive association was observed between UPF and VAT in this population (in line with our findings), the association between UPF and total fat mass is in contrast to our findings. These discrepancies may be partially explained by some key differences between our studies. Firstly, UPF was expressed in grams and not as %E. Secondly, the study population had a higher mean BMI, higher prevalence of T2DM, more smokers and fewer individuals with a higher education level compared to our study population. More importantly though, intake of fiber differed between tertiles of UPF at baseline, and through mediation analyses it was demonstrated that changes in fiber could explain 20% of the change in total fat mass. In addition, the three largest contributors to UPF at baseline were sweets, beverages (soft drinks and commercial juices) and processed meats. Lastly, it is noteworthy that the association between UPF and VAT in our population remained after adjustment for both dietary factors and BMI. Whether the associations between UPF and health outcomes are mediated by overall diet quality or not is actively debated, but overall, the associations appear to be robust when adjusting for both nutrients (e.g., saturated fat, sugars, sodium) and dietary patterns (e.g., Mediterranean diet) (32). The underlying mechanisms driving this association is unclear and warrants further investigation (33). Considering the heterogeneous nature of UPF, it may be that some, but not all, consumption patterns are associated with negative health outcomes as recently demonstrated for the incidence of type 2 diabetes (34). This concept requires further investigation to better understand the usefulness of UPF as an exposure variable in diet-health relationships.

Lastly, since all associations were attenuated after adjusting for potential confounders in model 1 (among them sex), we conducted *post hoc* analyses stratifying for sex. This was done due to observed differences in proportions of men and women among UPF-tertiles, as depicted in Table 1 and because sexual dimorphism in mechanistic pathways related to diet-induced liver fat accumulation (e.g., fatty acid oxidation and *de novo* lipogenesis) has been demonstrated in previous studies (35, 36). These *post hoc* analyses showed similar results as for the main regression models. Although associations for VAT and VAT/SAT were statistically significant for women and not for men, indicating a biological sex-diet interaction, findings from stratified *post hoc* analyses with lower statistical power should be interpreted with caution. Whether or not ectopic fat and VAT accumulation from UPF is sex-dependent is unclear and needs further investigation. However, a previous study based on dietary data from the National Health and Nutrition Examination Survey (NHANES) showed an interaction between being female and intake of UPF in relation to waist circumference (WC), a crude measure of VAT (3). Similar sex-specific associations for WC and abdominal obesity (dichotomized using WC) were recently demonstrated in a Korean population, further supporting our findings (37). Yet, in contrast to these findings, a recent prospective analysis indicated stronger association between intake of UPF and VAT in males compared to females (19).

In addition to the modest sample size, another limitation of the present work is the cross-sectional study design, making it impossible to draw any direct causal inferences from our findings. Another limitation commonly observed in nutritional epidemiology is the use of a FFQ to capture habitual dietary intake. Although a shorter version of the FFQ was previously validated against fatty acid biomarkers and weighed food records, FFQs are still prone to misreporting due to either inherent difficulties in remembering past diet exposure or due to social desirability bias. An increased participant burden may also contribute to misreporting if questionnaires are considered long and intricate. In addition, portion sizes were estimated based on age-specific usual intakes and may not reflect actual intakes. The restricted age-range may have led to lower variability in the exposure and outcomes, and hence contributed to the null findings on liver fat. An important strength of our study, however, is the use of MRI to assess visceral-, subcutaneous and ectopic fat mass. MRI-based assessment of ectopic fat depots, in particular liver fat, has been demonstrated to outperform other common non-invasive imaging modalities such as computed tomography (CT) and ultrasound when gold standard invasive histological grading has been used as reference (38). Another important strength is the population-based sample, which may increase generalizability of our findings. Furthermore, the ability to express the exposure of UPF as %E and not in % of grams is a strength. Although grams of UPF may better capture non-nutritive foods such as artificially sweetened beverages, human food consumption is more strongly associated with its caloric content than its weight and is therefore easier to interpret. Lastly, this study is the first population-based study that has investigated associations between intake of UPF and ectopic fat depots, thus providing important knowledge and possibly new hypotheses to this field.

In conclusion, energy intake from UPF is not associated with ectopic fat depots, SAT or total fat mass after adjustment for multiple confounders in this population having overall healthy food habits. However, a positive association between UPF and VAT was observed and this was driven by women. Whether lack of associations between UPF and the other fat depots could be partially explained by the lack of differences in SFA and total sugar intake and a higher intake of fiber in the highest tertile of UPF consumption warrants further investigation.

## Data Availability Statement

The raw data supporting the conclusions of this article will be made available by the authors, without undue reservation.

## Ethics Statement

The studies involving human participants were reviewed and approved by Ethics Committee of Uppsala University. The patients/participants provided their written informed consent to participate in this study.

## Author Contributions

FR, MF, and LL: designed research. JK, FR, and MF: conducted research. HA, JK, and LL: provided databases. FR, MF, and JK: analyzed data. MF and FR: wrote the article. FR: had primary responsibility for final content. All authors reviewed, revised, and accepted the final manuscript.

## Funding

MF was supported by the Swedish Diabetes Foundation, EXODIAB (Excellence of Diabetes Research in Sweden), FR was supported by the Swedish national strategic research initiative EXODIAB (Excellence of Diabetes Research in Sweden) and P.O. Zetterlings Foundation. HA and JK was supported by the Swedish Research Council (2016-01040). LL was supported by Uppsala University Hospital.

## Conflict of Interest

JK, HA were employed by Antaros Medical AB. The remaining authors declare that the research was conducted in the absence of any commercial or financial relationships that could be construed as a potential conflict of interest.

## Publisher's Note

All claims expressed in this article are solely those of the authors and do not necessarily represent those of their affiliated organizations, or those of the publisher, the editors and the reviewers. Any product that may be evaluated in this article, or claim that may be made by its manufacturer, is not guaranteed or endorsed by the publisher.
