# Supplementary material for: Intake of Ultra-Processed Food and Ectopic-, Visceral- and Other Fat Depots: A Cross-Sectional Study
Source: Front Nutr. 2022 Apr 4;9:774718. doi: 10.3389/fnut.2022.774718 (PMC9013765; doi:10.3389/fnut.2022.774718)
Supplement: Supplementary file 1 [file Data_Sheet_1.PDF]

# Supplementary Material

**Supplementary Table 1**. Characteristics of the study population stratified by men (n=133) and women (n=153)[a]

| | Men (n=133) | T1 of UPF (n=44)[b] | T2 of UPF (n=44) | T3 of UPF (n=45) | P-value (T3 vs T1) for men | Women (n=153) | T1 of UPF (n=51) | T2 of UPF (n=51) | T3 of UPF (n=51) | P-value (T3 vs T1) for women |
|---|---|---|---|---|---|---|---|---|---|---|
| Education (% 9y/10-12y/university) | 9/45/46 | 7/48/45 | 11/36/52 | 9/51/40 | P=0.6** | 2/46/52 | 0/31/69 | 2/55/43 | 4/53/43 | P=0.01** |
| Physical activity (% sedentary/light/moderate/high) | 16/21/31/33 | 9/16/28/47 | 21/19/26/35 | 18/27/38/18 | P=0.03 | 10/27/34/29 | 12/14/29/45 | 6/27/45/22 | 12/41/27/20 | P=0.007 |
| Current smoking (%) | 3 | 5 | 2 | 2 | P=0.62*** | 12 | 8 | 16 | 12 | P=0.51 |
| BMI (kg/m$^2$) | 26.1 (24.3-28.9) | 27.2 (24.2-29.8) | 25.6 (23.7-27.6) | 26.0 (24.4-29.5) | P=0.72 | 25.4 (22.7-28.4) | 24.3 (21.8-28.1) | 25.6 (23.2-29.0) | 26.0 (23.4-30.0) | P=0.15 |
| Liver fat (%) | 2.8 (1.7-6.3) | 2.3 (1.6-6.4) | 2.6 (1.4-5.3) | 3.9 (2.0-6.7) | P=0.66 | 1.5 (1.0-3.6) | 1.2 (0.9-2.1) | 1.8 (1.0-2.6) | 2.0 (1.1-6.1) | P=0.03 |
| NAFLD prevalence (n/%) | 38/29 | 13/30 | 10/23 | 15/33 | P=0.7 | 28/18 | 9/18 | 5/10 | 14/27 | P=0.24 |
| VAT (L) | 4.5 (2.8-5.9) | 4.5 (2.2-5.8) | 4.6 (2.8-6.1) | 4.6 (3.3-5.9) | P=0.83 | 2.1 (1.4-3.4) | 1.7 (1.0-3.2) | 2.2 (1.6-3.6) | 2.6 (1.7-3.7) | P=0.02 |
| Pancreas fat (%) | 5.1 (2.8-9.7) | 4.3 (2.2-9.9) | 5.5 (3.3-10.3) | 5.3 (3.3-9.2) | P=0.53 | 3.5 (2.2-5.7) | 3.0 (1.8-5.1) | 3.5 (2.1-6.3) | 4.4 (2.5-7.2) | P=0.19 |
| SAT (L) | 5.4 (3.9-7.2) | 5.2 (3.1-7.2) | 5.6 (3.7-7.9) | 5.4 (4.2-7.1) | P=0.36 | 7.0 (5.3-9.3) | 5.6 (4.0-8.7) | 7.4 (5.4-9.5) | 7.3 (5.7-9.1) | P=0.15 |
| VAT/SAT | 0.8 (0.6-1.0) | 0.8 (0.6-1.0) | 0.8 (0.6-0.9) | 0.8 (0.6-1.0) | P=0.99 | 0.3 (0.2-0.4) | 0.3 (0.2-0.3) | 0.3 (0.2-0.4) | 0.3 (0.3-0.4) | P=0.02 |
| Total fat mass (%) | 22.3±5.5 | 22.6±6.2 | 21.7±5.7 | 22.5±4.7 | P=0.87* | 33.8±7.3 | 32.9±7.7 | 34.2±7.3 | 34.2±7.1 | P=0.37 |
| Fasting plasma glucose (mmol/L) | 5.0 (4.6-5.4) | 5.0 (4.8-5.4) | 4.9 (4.6-5.4) | 4.9 (4.4-5.5) | P=0.19 | 5.0 (4.7-5.3) | 5.0 (4.7-5.2) | 5.1 (4.8-5.4) | 5.0 (4.6-5.3) | P=0.43 |
| Fasting serum insulin (mU/L) | 3.9 (2.7-6.1) | 3.8 (2.4-6.0) | 4.0 (2.3-5.5) | 3.7 (2.9-6.7) | P=0.51 | 3.4 (2.2-5.8) | 2.8 (1.8-4.9) | 3 (2.1-5.3) | 4.5 (2.9-7.8) | P=0.0009* |
| HOMA-IR | 0.9 (0.5-1.4) | 0.8 (0.5-1.4) | 0.9 (0.5-1.2) | 0.9 (0.6-1.6) | P=0.64* | 0.7 (0.5-1.3) | 0.6 (0.4-1.1) | 0.7 (0.5-1.2) | 1.0 (0.6-1.9) | P=0.001* |

| | | | | | | | | | | |
|---|---|---|---|---|---|---|---|---|---|---|
| Total cholesterol (mmol/L) | 5.4±1.1 | 5.6±1.4 | 5.3±1.0 | 5.4±0.9 | P=0.33 | 5.2±0.9 | 5.1±0.8 | 5.2±0.9 | 5.2±0.9 | P=0.88* |
| LDL-cholesterol (mmol/L) | 3.6±2.9 | 3.7±1.1 | 3.4±0.9 | 3.6±0.8 | P=0.74 | 3.2±0.8 | 3.2±0.7 | 3.2±0.8 | 3.3±0.8 | P=0.65 |
| HDL-cholesterol (mmol/L) | 1.2 (1.1-1.4) | 1.2 (1.1-1.4) | 1.3 (1.1-1.4) | 1.2 (1.1-1.4) | P=0.52 | 1.5 (1.3-1.7) | 1.5 (1.3-1.8) | 1.5 (1.4-1.7) | 1.4 (1.2-1.6) | P=0.25 |
| TG (mmol/L) | 1.2 (0.8-1.6) | 1.2 (0.8-1.6) | 1.1 (0.8-1.6) | 1.1 (0.8-1.5) | P=0.83 | 0.9 (0.6-1.2) | 0.8 (0.6-1.2) | 0.9 (0.6-1.3) | 1.0 (0.8-1.3) | P=0.03 |
| NOVA group 1 (%E) | 39.7±9.7 | 48.2±8.6 | 39.1±6.5 | 32.0±6.1 | P<0.0001 | 47.1±10.2 | 53.8±10.7 | 48.1±7.7 | 39.3±5.8 | P<0.0001 |
| NOVA group 2 (%E) | 0.0 (0.0-0.3) | 0.1 (0.0-0.5) | 0.0 (0.0-0.6) | 0.0 (0.0-0.2) | P=0.25 | 0.0 (0.0-0.4) | 0.0 (0.0-0.2) | 0.0 (0.0-0.6) | 0.1 (0.0-0.6) | P=0.61 |
| NOVA group 3.1 (%E) | 17.9±6.5 | 19.8±7.2 | 18.3±5.8 | 15.5±5.7 | P=0.004 | 18.3±8.1 | 21.7±10.0 | 17.5±7.2 | 15.6±5.2 | P=0.003* |
| NOVA group 3.2 (%E) | 42.0±9.5 | 31.4±5.0 | 42.2±2.6 | 52.2±4.8 | P<0.0001 | 34.1±9.3 | 24.0±4.6 | 33.9±2.4 | 44.2±5.5 | P<0.0001 |
| Energy (kcal) | 2714.6±639.4 | 2479.6±600.2 | 2840.8±662.4 | 2821.1±602.5 | P=0.009 | 1884.3±455.6 | 1861.9±449.7 | 1909.7±476.1 | 1881.2±448.2 | P=0.83 |
| Carbohydrates (%E) | 41.6±5.9 | 38.9±6.6 | 41.5±4.9 | 44.2±5.2 | P<0.0001 | 41.9±7.4 | 39.0±8.4 | 42.3±7.1 | 44.3±5.7 | P=0.002* |
| Fiber (g) | 32.2 (24.9-38.5) | 29.7 (22.8-36.3) | 34.2 (25.5-41.3) | 32.2 (26.8-38.7) | P=0.11 | 24.2 (18.8-28.9) | 25.2 (19.1-29.6) | 23.2 (17.7-27.9) | 23.5 (18.0-29.5) | P=0.48 |
| Total sugar (%E) | 13.9 (11.8-16.2) | 14.5 (11.8-16.3) | 13.7 (12.5-16.9) | 14.2 (11.1-15.9) | P=0.55 | 15.2 (13.0-18.7) | 16.1 (12.4-18.7) | 15.2 (13.1-18.7) | 15.1 (13.0-19.9) | P=0.68 |
| Fat (%E) | 34.7±5.1 | 35.9±6.6 | 34.8±4.3 | 33.5±3.9 | P=0.08 | 34.1±6.1 | 34.9±7.5 | 34.1±5.8 | 33.5±4.6 | P=0.54* |
| SFA (%E) | 14.7±2.9 | 15.2±3.5 | 14.8±2.6 | 14.1±2.6 | P=0.11 | 14.1±3.2 | 14.2±3.7 | 14.2±3.2 | 13.9±2.8 | P=0.91 |
| MUFA (%E) | 11.6 (10.8-12.7) | 12.1 (10.9-13.2) | 11.7 (11.1-12.8) | 11.4 (10.0-12.5) | P=0.07 | 11.6 (10.2-13.0) | 11.2 (10.1-13.2) | 11.6 (10.3-13.0) | 11.7 (10.3-13.1) | P=0.82* |
| PUFA (%E) | 5.0 (4.3-5.7) | 4.8 (4.3-5.9) | 5.1 (4.3-5.7) | 5.0 (4.4-5.7) | P=0.74 | 5.2 (4.3-6.2) | 5.6 (4.2-6.9) | 5.1 (4.5-6.1) | 4.9 (4.3-5.8) | P=0.04 |
| Protein (%E) | 18.3±2.3 | 19.7±2.0 | 18.1±2.2 | 17.2±1.9 | P<0.0001 | 18.7±2.9 | 20.2±3.2 | 18.5±2.4 | 17.3±2.1 | P<0.0001 |
| Alcohol (%E) | 2.5 (1.1-3.9) | 2.9 (1.5-4.3) | 2.7 (1.4-4.5) | 1.9 (0.7-3.5) | P=0.08 | 2.2 (0.7-3.5) | 2.5 (1.2-4.0) | 1.6 (0.6-2.7) | 2.3 (0.4-3.4) | P=0.05 |
| HDI | 1.8±0.9 | 1.8±1.0 | 1.7±0.8 | 1.8±1.0 | P=0.38* | 2.4±1.3 | 2.5±1.2 | 2.4±1.2 | 2.1±1.4 | P=0.09 |

[a]Data are presented as mean±SD, %, counts or as median (IQR) for skewed distributed variables.

[b]Tertile 1 indicates the lowest proportion of UPF-intake while tertile 3 indicates the highest proportion of UPF-intake.

BMI, Body Mass Index; HDI, healthy diet indicator; HDL, High-Density Lipoprotein; HOMA-IR, Homeostatic Model Assessment of Insulin Resistance; LDL, Low-Density Lipoprotein; MUFA, monounsaturated fat; NAFLD, Non-alcoholic fatty liver disease; PUFA, Polyunsaturated Fatty Acids; SFA, Saturated Fatty Acids; T, Tertile; TG, Triglycerides; UPF, Ultra-processed food; VAT, Visceral Adipose Tissue.
NOVA group 1, unprocessed or minimally processed foods; NOVA group 2, processed culinary ingredients; NOVA group 3.1, processed foods; NOVA group 3.2, ultra-processed foods.
*Analyzed non-parametrically using the Mann-Whitney U test.
**9y + 10-12y categories were combined to satisfy the assumption of the chi-squared test (at least 80% of the expected frequencies are greater than or equal to 5). Categories 9y + 10-12y were combined for both men and women in order for them to be easier compared to each other although only women had >20% of the expected frequencies less than 5.
***Analyzed non-parametrically using the Fisher´s exact test.

**Supplementary Table 2**. Energy contribution from food items within the ultra-processed food (UPF) category[a]

| UPF food item | Energy (kcal) All (n=286) | Energy (kcal) Men (n=133) | Energy (kcal) Women (n=153) |
|---|---|---|---|
| Crisp bread | 74.3 (31.9-148.7) | 107.1 (45.9-214.2) | 53.1 (21.2-148.7) |
| Whole grain bread | 56.9 (11.4-57.8) | 90.2 (22.5-157.8) | 45.5 (11.4-79.6) |
| Butter/margarine as spread on Sandwich | 49.8 (22.3-97.6) | 71.2 (30.5-128.2) | 43.0 (20.8-85.4) |
| Pizza | 37.5 (37.5-42.3) | 42.3 (42.3-42.3) | 37.4 (0.0-37.4) |
| Chocolate | 35.9 (11.2-41.2) | 41.2 (12.8-41.2) | 35.9 (11.2-35.9) |
| Buns/cookies | 25.4 (7.9-39.3) | 39.3 (12.2-39.3) | 25.4 (7.9-25.4) |
| Fiber enriched bread | 24.1 (0.0-93.6) | 70.2 (0.0-163.7) | 24.1 (0.0-66.2) |
| Pancakes/crepes | 23.6 (0.0-31.1) | 31.1 (0.0-31.1) | 23.6 (0.0-23.6) |
| Chips/popcorn/cheese puffs | 22.8 (10.1-32.5) | 22.8 (22.8-48.1) | 10.1 (10.1-32.5) |
| Other sausage | 15.8 (0.0-21.1) | 21.1 (0.0-21.1) | 15.8 (0.0-15.8) |
| Sausage (falukorv) | 14.6 (0.0-19.6) | 19.6 (19.6-19.6) | 14.6 (0.0-14.6) |
| Cakes/pastries | 13.5 (0.0-18.3) | 18.3 (0.0-18.3) | 13.5 (0.0-13.5) |
| Ice cream | 12.5 (9-29) | 12.5 (12.5-40.2) | 9.0 (9.0-9.0) |
| White bread/limpa | 10.5 (0.0-57.4) | 38.2 (0.0-95.6) | 0.0 (0.0-21.2) |
| Müesli | 10.2 (0.0-48.1) | 10.2 (0.0-76.3) | 6.4 (0.0-48.1) |
| Candy (not chocolate) | 9.3 (4.8-15.5) | 9.3 (9.3-30.0) | 4.8 (4.8-15.5) |
| Mayonnaise | 7.7 (0.0-11.3) | 7.7 (0.0-7.7) | 0 (0-11.3) |
| Biscuits/wafers | 7.5 (0.0-15.7) | 7.5 (0.0-24.1) | 4.9 (0.0-15.7) |
| French fries | 6.3 (0.0-14.3) | 14.3 (14.3-14.3) | 6.3 (0.0-6.3) |
| Other jam | 4.9 (0.0-6.1) | 6.1 (0.0-6.1) | 4.9 (0.0-4.9) |
| Cold cuts sausage (e.g. salami) | 4.9 (0.0-15.6) | 8.3 (8.3-26.7) | 4.8 (0.0-4.8) |
| Lingonberry jam | 4.7 (3.3-4.7) | 4.7 (4.7-4.7) | 3.3 (3.3-7.0) |
| Salad dressing | 3.9 (0.0-12.7) | 12.3 (0.0-39.5) | 3.9 (0.0-3.9) |
| Swedish caviar (e.g. Kalles kaviar) | 3.4 (0.0-10.8) | 4.0 (0.0-13.0) | 3.4 (0.0-10.8) |
| Ketchup | 2.6 (0.0-6.3) | 2.6 (0-8.4) | 2.0 (0.0-6.3) |
| Spirits | 1.6 (0.0-8) | 2.6 (2.6-13.2) | 0.0 (0.0-1.6) |
| Fruit yogurt/sour milk | 0.0 (0.0-27.5) | 0.0 (0.0-55.0) | 0.0 (0.0-10.5) |
| Blood pudding/sausage | 0.0 (0.0-25.5) | 0.0 (0.0-25.5) | 0.0 (0.0-27.2) |
| Pea soup | 0.0 (0.0-19.8) | 19.8 (0.0-19.8) | 0.0 (0.0-13.1) |
| Liver paté | 0.0 (0.0-18.4) | 0.0 (0.0-9.8) | 0.0 (0.0-18.4) |
| Other soda/fruit juice (saft) | 0.0 (0.0-12.2) | 0.0 (0.0-18.7) | 0.0 (0.0-12.2) |
| Breakfast cereal | 0.0 (0.0-10.5) | 0.0 (0.0-10.5) | 0.0 (0.0-6.6) |
| Lean sausage | 0.0 (0.0-8) | 0.0 (0.0-11.4) | 0.0 (0.0-8.0) |
| Liqueur/ sherry/ liqueur | 0.0 (0.0-1.4) | 0.0 (0.0-2.7) | 0.0 (0.0-1.4) |
| Salad dressing (reduced fat/fat free) | 0.0 (0.0-0.8) | 0.0 (0.0-2.4) | 0.0 (0.0-0.8) |
| Coca Cola/Pepsi, light | 0.0 (0.0-0.0) | 0.0 (0.0-0.0) | 0.0 (0.0-0.0) |
| Coca Cola/Pepsi | 0.0 (0.0-0.0) | 0.0 (0.0-20.1) | 0.0 (0.0-0.0) |

| | | | |
|---|---|---|---|
| Other soda/fruit juice (saft), light | 0.0 (0.0-0.0) | 0.0 (0.0-0.0) | 0.0 (0.0-0.0) |
| Cream cheese (low-fat) | 0.0 (0.0-0.0) | 0.0 (0.0-0.0) | 0.0 (0.0-0.0) |
| Cream cheese | 0.0 (0.0-0.0) | 0.0 (0.0-0.0) | 0.0 (0.0-0.0) |
| Liver paté (low-fat) | 0.0 (0.0-0.0) | 0.0 (0.0-0.0) | 0.0 (0.0-0.0) |
| Fish sticks | 0.0 (0.0-0.0) | 0.0 (0.0-9.1) | 0.0 (0.0-0.0) |
| Fruit fool/ fruit soup | 0.0 (0.0-0.0) | 0.0 (0.0-0.0) | 0.0 (0.0-0.0) |
| Mayonnaise (reduced fat/fat free) | 0.0 (0.0-0.0) | 0.0 (0.0-3.5) | 0.0 (0.0-0.0) |

[a]Data are presented as median (IQR).

**Supplementary Table 3.** Post-hoc sensitivity linear regression analyses between ultra-processed food (%E) excluding whole-grain bread and crisp bread and fat depots[a]

| | **Crude** β (95% CI) | **P** | **Model 1[b]** β (95% CI) | **P** | **Model 2[c]** β (95% CI) | **P** | **Model 3[d]** β (95% CI) | **P** |
|---|---|---|---|---|---|---|---|---|
| **Ln liver fat** | 0.02 (0.005, 0.03) | 0.006 | 0.002 (-0.01, 0.01) | 0.78 | 0.01 (-0.002, 0.03) | 0.09 | 0.01 (-0.0001, 0.02) | 0.05 |
| **Ln pancreas fat** | 0.01 (0.003, 0.02) | 0.01 | 0.004 (-0.01, 0.02) | 0.41 | 0.01 (-0.003, 0.02) | 0.16 | 0.01 (-0.003, 0.02) | 0.15 |
| **Ln VAT** | 0.02 (0.01, 0.03) | 0.0004 | 0.001 (-0.01, 0.01) | 0.82 | 0.01 (-0.004, 0.02) | 0.26 | 0.01 (-0.001, 0.01) | 0.07 |
| **Ln SAT** | -0.0005 (-0.01, 0.01) | 0.89 | -0.0002 (-0.01, 0.01) | 0.62 | 0.001 (-0.01, 0.01) | 0.86 | 0.001 (-0.003, 0.01) | 0.54 |
| **Ln VAT/SAT** | 0.02 (0.01, 0.03) | <0.0001 | 0.003 (-0.003, 0.01) | 0.32 | 0.005 (-0.001, 0.01) | 0.11 | 0.01 (-0.001, 0.01) | 0.10 |
| **Total fat mass** | -0.14 (-0.25, -0.04) | 0.008 | -0.03 (-0.11, 0.05) | 0.47 | -0.03 (-0.13, 0.06) | 0.51 | -0.03 (-0.09, 0.02) | 0.27 |

[a]Data are presented as β with 95% CI and corresponding P-values.
SAT, subcutaneous adipose tissue; VAT, visceral adipose tissue.
n(liver fat) = 282-286, n(pancreas fat) = 276-279 , n(VAT, SAT, VAT/SAT) = 238-241, n(Total fat mass) = 281-285.
[b]Adjusted for sex, education, physical activity and current smoking status.
[c]Adjusted for Model 1 + protein, fiber, alcohol, saturated fat, polyunsaturated fat and total sugar.
[d]Adjusted for Model 2 + BMI (Body mass index).

**Supplementary Table 4**. Sex-stratified linear regression analyses between ultra-processed food and fat depots[a]

| | Crude β (95% CI) | P | Model 1 β (95% CI)[b] | P | Model 2 β (95%CI)[c] | P | Model 3 β (95% CI)[d] | P |
|---|---|---|---|---|---|---|---|---|
| **Ln liver fat** | | | | | | | | |
| Men | 0.001 (-0.02, 0.02) | 0.90 | -0.01 (-0.03, 0.01) | 0.18 | 0.001 (-0.02, 0.02) | 0.92 | -0.004 (-0.02, 0.01) | 0.68 |
| Women | 0.02 (-0.001, 0.03) | 0.06 | 0.01 (-0.01, 0.02) | 0.53 | 0.02 (-0.004, 0.04) | 0.12 | 0.02 (-0.003, 0.03) | 0.10 |
| **Ln pancreas fat** | | | | | | | | |
| Men | 0.01 (-0.01, 0.02) | 0.43 | 0.002 (-0.01, 0.02) | 0.82 | 0.01 (-0.01, 0.03) | 0.35 | 0.005 (-0.01, 0.02) | 0.61 |
| Women | 0.01 (-0.01, 0.02) | 0.38 | 0.004 (-0.01, 0.02) | 0.58 | 0.01 (-0.004, 0.03) | 0.12 | 0.01 (-0.005, 0.03) | 0.18 |
| **Ln VAT** | | | | | | | | |
| Men | 0.01 (-0.003, 0.02) | 0.14 | 0.002 (-0.01, 0.01) | 0.77 | 0.004 (-0.01, 0.02) | 0.61 | 0.001 (-0.01, 0.01) | 0.90 |
| Women | 0.01 (0.001, 0.02) | 0.04 | 0.01 (-0.005, 0.02) | 0.21 | 0.02 (0.001, 0.03) | 0.04 | 0.01 (0.003, 0.02) | 0.01 |
| **Ln SAT** | | | | | | | | |
| Men | 0.01 (-0.003, 0.02) | 0.19 | 0.002 (-0.01, 0.01) | 0.72 | 0.01 (-0.01, 0.02) | 0.29 | 0.003 (-0.004, 0.01) | 0.38 |
| Women | 0.01 (-0.003, 0.01) | 0.23 | -0.0002 (-0.01, 0.01) | 0.97 | 0.003 (-0.01, 0.01) | 0.54 | 0.001 (-0.005, 0.01) | 0.72 |
| **Ln VAT/SAT** | | | | | | | | |
| Men | 0.002 (-0.004, 0.01) | 0.55 | -0.0001 (-0.01, 0.01) | 0.97 | -0.003 (-0.01, 0.01) | 0.48 | -0.003 (-0.01, 0.01) | 0.49 |
| Women | 0.01 (-0.00001, 0.01) | 0.05 | 0.01 (0.0002, 0.02) | 0.04 | 0.01 (0.003, 0.02) | 0.01 | 0.01 (0.003, 0.02) | 0.01 |
| **Total fat mass** | | | | | | | | |
| Men | 0.001 (-0.10, 0.10) | 0.98 | -0.07 (-0.16, 0.03) | 0.18 | -0.002 (-0.12, 0.11) | 0.98 | -0.05 (-0.13, 0.02) | 0.13 |
| Women | 0.05 (-0.08, 0.17) | 0.47 | -0.004 (-0.14, 0.13) | 0.95 | -0.04 (-0.20, 0.13) | 0.66 | -0.06 (-0.16, 0.04) | 0.24 |

[a]Data are presented as β with 95% CI and corresponding P-values.
SAT, subcutaneous adipose tissue; VAT, visceral adipose tissue.
n(liver fat men) = 131-133, n(liver fat women) = 151-153, n(pancreas fat men) = 129-131, n(pancreas fat women) = 147-148, n(VAT, SAT, VAT/SAT men) = 101-103, n(VAT, SAT, VAT/SAT women) = 137-138, n(Total fat mass men) = 131-133, n(Total fat mass women) = 150-152.
[b]Adjusted for education, physical activity and current smoking status.
[c]Adjusted for Model 1 + protein, fiber, alcohol, saturated fat, polyunsaturated fat and total sugar.
[d]Adjusted for Model 2 + BMI (Body mass index).
